# Tobacco control and household tobacco consumption: A tale of two educational groups

**DOI:** 10.1002/hec.4122

**Published:** 2020-06-21

**Authors:** Biplab Kumar Datta, Muhammad Jami Husain, Ishtiaque Fazlul

**Affiliations:** ^1^ Global Noncommunicable Diseases Branch, Division of Global Health Protection Center for Global Health, Centers for Disease Control and Prevention Atlanta Georgia USA; ^2^ Department of Economics Georgia State University Atlanta Georgia USA

**Keywords:** inequality, population health, public health, smoking

## Abstract

Since the ratification of the World Health Organization Framework Convention on Tobacco Control in 2004, Pakistan has made modest but continued progress in implementing various tobacco control measures. By 2014, substantial progress was achieved in areas of monitoring, mass media antitobacco campaigns, and advertising bans. However, the findings from the 2014 Global Adult Tobacco Survey of Pakistan show significant differences in antitobacco campaign exposure among individuals of different educational attainment. Given this large variation in noticing antitobacco information, this paper analyzes how heterogeneity in treatment exposure may differentially impact tobacco‐use prevalence across household groups. Household‐level tobacco‐use prevalence in 2014 was, respectively, 56% and 48% for the low‐ and high‐education households. The gap in tobacco‐use prevalence between the two educational groups further widens post 2014. We find that, on average, individuals with higher than primary education are 14 percentage points and 6 percentage points more likely to notice anticigarette and antismokeless tobacco information in 2014, respectively. Subsequently, in 2016, high‐education households experienced a 3.6 percentage point higher reduction in tobacco‐use prevalence compared to the low‐education households. These findings motivate policies to enhance the outreach of tobacco control measures across different educational groups.

## 1 INTRODUCTION

### 1.1 Background

The World Health Organization (WHO) Framework Convention on Tobacco Control (FCTC) is the first global evidence‐based public health treaty to combat the global tobacco epidemic and protect people from the negative consequences of tobacco use. In line with the WHO FCTC, MPOWER package, a set of six policy guidelines, was introduced to “assist in the country‐level implementation of effective measures to reduce the demand for tobacco” (World Health Organization, 2009). Two of the main recommendations of FCTC include the requirement of health warning messages and pictures on tobacco product packaging and the use of mass media campaigns in television, radio, print, and other media outlets. The corresponding FCTC Articles 11 and 12, respectively, recommend use of large pictorial health warnings and removal of misleading information such as “mild” and “light” on tobacco products and educating people about the health, social, and environmental consequences of tobacco production and consumption (World Health Organization, 2003).

### 1.2 Existing evidence

As recommended in FCTC Article 11, health warnings on tobacco packages have been adopted in many developed and developing countries. The literature on the effectiveness of health warnings suggests that warning labels facilitate improved risk perception of tobacco use and promote cessation behavior in several developed countries (Borland et al., 2009; Hammond, Fong, McNeill, Borland, & Cummings, 2006; Hammond, Wakefield, Durkin, & Brennan, 2012; Kuehnle, 2019). Size of the warnings matters (Hammond, 2011) and pictorial warnings are found more effective than text (Noar et al., 2016), especially in the population with lower levels of educational attainment (Thrasher et al., 2010). Though most of the experimental and market research evidence on the effect of health warnings on tobacco packaging is from developed countries, there is some evidence from developing countries that such health warnings reach the target audience (Alaouie, Afifi, Haddad, Mahfoud, & Nakkash, 2015; Andreeva & Krasovsky, 2011; Yong et al., 2013). A recent study also indicates that the effectiveness of pictorial warnings is generally consistent across developed and developing countries (Hammond et al., 2019).

FCTC Article 12 entails using a variety of methods and media vehicles to curb the demand for tobacco products. Mass media campaigns are found effective to reduce the take‐up of smoking and to promote quitting in developed countries (Durkin, Brennan, & Wakefield, 2012; National Cancer Institute, 2008). Research also finds the complementarity of mass media campaigns with other tobacco control strategies, such as increases in tobacco taxation or smoke‐free policies (Azagba, Burhoo, Chaloupka, & Fong, 2015; Wakefield et al., 2008). The evidence on the effect of mass media campaigns on the disparity in smoking prevalence between high and low socioeconomic status (SES), however, is inconclusive (Niederdeppe, Kuang, Crock, & Skelton, 2008). In developing country settings, few studies find the effectiveness of antitobacco campaigns in terms of increased knowledge about harmful health effects (Jin, Song, & Zhang, 2018) and being concerned about the tobacco use habit (Murukutla et al., 2012). Yet little is known about the impact of mass media antitobacco campaigns on tobacco consumption in low‐ and middle‐income countries (LMICs).

### 1.3 The Pakistan context

**FIGURE 1:**
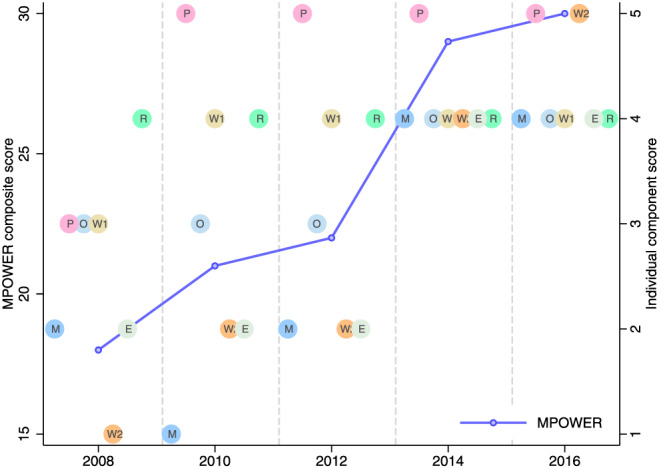
Trend in MPOWER score in Pakistan. MPOWER score is the aggregate of attainment levels of *M*, *P*, *O*, *W*, and *R*. *W*_1_ and *W*_2_, respectively, refer to health warnings and mass media antitobacco campaigns. The lowest attainment level is scored as 1, and the highest attainment level is scored as 4 for *M* and 5 for the rest of the measures. The composite MPOWER score, therefore, can range from a minimum of 7 to a maximum of 34. The attainment levels are obtained from the WHO Report on the Global Tobacco Epidemic, various issues [Colour figure can be viewed at wileyonlinelibrary.com]

Since the ratification of the WHO FCTC in 2004, Pakistan, a lower‐middle‐income country, made gradual progress in implementing the MPOWER package. Change in MPOWER composite score (an indicator of the implementation level) in Pakistan was modest during the 2008 to 2012 period but got a big boost from 2012 to 2014 (Figure 1). This big jump in 2014 was largely due to the progress achieved in *M* (i.e., availability of representative and periodic data of smoking prevalence for both adults and youth), *W*_2_ (i.e., national antitobacco campaign conducted with five to six appropriate characteristics), and *E* (i.e., bans on tobacco advertising, promotion, and sponsorship on national television, radio, and print media) components of the MPOWER package. Following the progress achieved in areas of monitoring, mass media antitobacco campaigns, and advertising bans, the share of tobacco‐consuming households decreased by more than 6 percentage points in 2016, compared to that in 2014. However, the findings from the 2014 Global Adult Tobacco Survey (GATS) of Pakistan revealed significant differences in antitobacco campaign exposure among individuals of different educational attainment (Pakistan Health Research Council, 2016). For instance, only 27% of the adult (15+ age) individuals with educational attainment of “primary or less” noticed any anticigarette information in 2014, whereas for adults with higher than primary education, that share was as high as 53%. Given this large variation in the exposure to antitobacco information, the question arises whether the decrease in household‐level tobacco use from 2014 to 2016 is disproportionate across households of different education levels.

### 1.4 Study purpose

Based on the findings of the 2014 GATS, we hypothesize that treatment exposure of MPOWER interventions in Pakistan was greater for households with a higher level of adult education. The variations in noticing antitobacco information in mass media or health warnings in tobacco packaging by respondents' education level, estimated from the 2014 GATS, provide a unique opportunity to analyze the impact of MPOWER implementation on tobacco‐use prevalence across socioeconomic groups in Pakistan. Previous studies on MPOWER and tobacco use mainly focus on cross‐country variations in MPOWER implementation and its impact on adult smoking prevalence (Dubray, Schwartz, Chaiton, O'connor, & Cohen, 2015; Gravely et al., 2017; Levy, Yuan, Luo, & Mays, 2018; Ngo, Cheng, Chaloupka, & Shang, 2017). We contribute to the literature by investigating how the heterogeneity in treatment exposure (by education group) may impact outcomes (e.g., tobacco‐use prevalence) of MPOWER implementation within a country.

Education is presumed to be an important determinant of an individual's tobacco‐use behavior. The prevalence of tobacco use is inversely associated with an individual's level of education in LMICs (Bosdriesz, Mehmedovic, Witvliet, & Kunst, 2014; Palipudi et al., 2012; Sreeramareddy, Harper, & Ernstsen, 2018). This pattern is also evident in the findings of GATS and household income and expenditure survey of Pakistan in 2014. At the individual level, the tobacco‐use prevalence was 23% for lesseducated (primary or less) individuals, 10 percentage points higher than that of more educated (higher than primary) individuals. At the household level, 56% of the lesseducated households (highest adult education of primary or less) consumed tobacco products, which was 8 percentage points higher than that for more educated households (highest adult education of higher than primary). Given this existing gap in tobacco use between the two educational groups, our impact analysis is thus centered on examining whether the gap (at the household level) converges (narrows) or diverges (widens) post 2014.

We use 2012, 2014, and 2016 versions of the household income and expenditure surveys (household‐integrated economic survey) in Pakistan and estimate a difference‐in‐differences (DD) model. We find evidence of a larger decrease in tobacco‐use prevalence for households with a greater level of education, meaning widening of the tobacco‐use prevalence gap between low and high educational groups post 2014. Our study thus contributes to the literature in two major ways. First, exploiting the differences in treatment exposure across educational groups, we provide an estimate of the within‐country impact of MPOWER implementation in a developing country setting. Second, our results provide important insights on the necessity of designing inclusive antitobacco campaigns that can effectively reach individuals of different levels of educational attainment.

## 2 METHODS

### 2.1 MPOWER measures

MPOWER package consists of six measures—*M*: monitor tobacco use; *P*: protect people from tobacco smoke; *O*: offer help to quit tobacco use; *W*: warn about the dangers of tobacco; *E*: enforce bans on tobacco advertising and promotion; and *R*: raise taxes on tobacco products (World Health Organization, 2017). The *W* measure has two components: health warnings on tobacco packaging (*W*_1_) and mass media antitobacco campaigns (*W*_2_). The WHO has been systematically tracking and reporting the country‐level implementation of MPOWER intervention by each measure. Countries are scored for each measure based on their attainment level of that measure. The lowest attainment level is scored as 1, and the highest attainment level is scored as 4 for *M* and 5 for the other measures. A composite score generated from aggregating a country's MPOWER measure scores can serve as an indicator of the country's MPOWER implementation status (Dubray et al., 2015; Ngo et al., 2017). The higher the score, the higher is the progress in MPOWER implementation.

The WHO reported increase in the *M*, *O*, *W*_2_, and *E* scores from 2012 to 2014 in Pakistan (Figure 1). Monitoring tobacco use to generate prevalence data mainly informs the administering of tobacco control initiatives rather than discouraging tobacco use at the individual level. The bans on advertising of tobacco products are supposed to confine individuals' exposure to tobacco product promotion regardless of their educational attainment. The progress in the *M* and *E* measures, in general, would not have differential exposures across educational groups. There was a slight improvement in the *O* measure (i.e., costs of nicotine replacement therapy or some other cessation services are covered), but the coverage of cessation services in Pakistan is very limited (Gilani & Leon, 2013; Khan, 2012). This is not uncommon in many LMICs (Piné‐Abata et al., 2013). Of the 224 individuals in GATS, who reported to have tried to stop smoking during the past 12 months, only 7% reported having nicotine replacement therapy, and only 14% reported receiving counseling at a smoking cessation clinic. The small coverage of cessation programs combined with modest progress in *O* would have minimal differential impacts across different socioeconomic groups. The differential impact (across educational groups) of progress in MPOWER implementation in Pakistan, hence, is mostly attributable to the progress in the *W*_2_ measure.

### 2.2 Data

We used household‐level data from the nationally representative 2011–2012, 2013–2014, and 2015–2016 cohorts of the Household Integrated Economic Survey (HIES) of Pakistan. We defined household education levels as *primary or no education* if none of the adult (15+ age) members of the household attained education beyond the elementary school (Grade 5) or *higher than primary education* if at least one adult (15+ age) member attained higher than primary, that is, secondary (middle/high) school or higher level of education. The HIES 2011–2012, 2013–2014, and 2015–2016 cohorts surveyed 15,807, 17,991, and 24,238 households in respective survey years. Around 60% of the households in all surveys are *higher than primary* type households.

HIES collects information on households' monthly consumption expenditure on cigarettes, biri, chewing tobacco (including saunf, niswar, and gutka), betel leaves, and betel nuts. A household is categorized as a tobacco‐user household if that household reports positive monthly expenditure on any of these tobacco products. Tobacco‐user households, on average, spent 2.6% (in 2011–2012 and 2013–2014) to 3.0% (in 2015–2016) of monthly expenditure on tobacco products. Share of tobacco‐user households was around 50% during 2011–2012 and 2013–2014, and it decreased by around 6 percentage points in 2015–2016.

We use data from the Pakistan GATS 2014, a nationally representative survey of 7,831 individuals to analyze individual‐level exposure to antitobacco campaigns. Around 40% of the respondents in GATS 2014 reported educational attainment of higher than primary. The GATS asks questions on whether the respondent noticed any information about the dangers of tobacco use or information that encourages quitting tobacco. It also asks whether the respondent noticed any health warnings on tobacco product packaging (e.g., cigarette packages). These allow us to measure the reach of antitobacco campaigns in 2014 across individuals of different educational attainment. Both the HIES and GATS surveys cover representative urban and rural households from the 27 administrative divisions of Pakistan.

### 2.3 Linear probability model

We first run a linear probability model (LPM) regressions using HIES 2013–2014 and 2015–2016 data to estimate the changes in the share of tobacco‐user households from 2014 to 2016 for the two educational groups. We also run logistic regression specification, but the average marginal effect estimates of the logistic regression are not much different from estimates of the linear model. We perform the Chow test to examine whether the estimated coefficients for the two educational groups are different. The LPM specification, separately estimated for all households, and for *primary or no education* and *higher than primary education* households, is as follows:

(1) $$\text{Tobacc}o_{i}=\alpha_{0}+\alpha_{1}\mathrm{Yea}r_{2016,i}+\alpha_{2}\text{Urba}n_{i}+X_{i}\alpha_{3}+\text{Divisio}n_{i}+\upsilon_{i},$$

where *Tobacco*_*i*_ is a dummy that takes the value 1 if household *i* consumes any tobacco products and 0 otherwise. *Year*_2016,*i*_ is a dummy that takes the value 1 if the year is 2016, and 0 if the year is 2014. We assume that tobacco consumption in year *t* is impacted by tobacco control measures in year *t–k*, where *k* is the policy transmission period. The impact of tobacco control measures in 2014, therefore, is not visible in tobacco consumption of 2014; rather, the impact is assumed to be observed in 2016 tobacco consumption. The coefficient *α*_1_ measures the change in share of tobacco‐user households post 2014. A negative (<0) estimate of *α*_1_ indicates a decrease in tobacco‐user household share from 2014 to 2016. *Urban*_*i*_ is a dummy that takes the value 1 if household *i* is located in urban areas and 0 if located in rural areas. ***X*** is a vector of household‐level characteristics including log of household income per capita (in constant 2008 prices), the share of adults (age 15+) in the household, the share of male among adults in the household, the share of elderly (age 65+) in the household, sex of the household head, and dummies for household size. These are standard control variables in the household tobacco‐use literature (John, Ross, & Blecher, 2012; Wang, Sindelar, & Busch, 2006). *Division*_*i*_ controls for division fixed effect (such as regional differences in antitobacco campaign administration, social norms, and tobacco price elasticities), and *υ*_*i*_ is the idiosyncratic error term. Errors are clustered at the division level. If estimates of *α*_1_ are found different for the two educational groups, it indicates the potential differential impact of MPOWER implementation across the two education groups.

### 2.4 Exposure to antitobacco campaign

Next, we analyze the GATS data to generate evidence on differential exposure of antitobacco campaigns in 2014 across educational groups. We run the following LPM specification:

(2) $$\text{Exposur}e_{j}=\gamma_{0}+\gamma_{1}\mathrm{Edu}c_{j}+{\mathrm{DC}}_{j}\gamma_{3}+{\mathrm{HC}}_{j}\gamma_{4}+\text{Urba}n_{j}+\text{Divisio}n_{j}+\zeta_{j},$$

where *Exposure*_*j*_ is the dummy that indicates whether individual *j* was exposed to certain antitobacco measures including anticigarette smoking information (anywhere, on radio/television or on billboards), antismokeless tobacco information (anywhere, on radio/television or on billboards), health warning on cigarette packages, and health warning on smokeless tobacco products. Separate regressions are run for exposure to each of the antitobacco measures. *Educ*_*j*_ is a dummy that takes the value 1 if individual *j*'s level of education is beyond primary (i.e., Grade 6 and above), 0 otherwise. A statistically significant estimate of *γ*_*1*_, the coefficient of *Educ*_*j*_, refers to differences in antitobacco campaign exposure across individuals of *primary or no education* and *higher than primary education.* ***DC*** is a vector of demographic characteristics including gender, age, marital status, and occupation. ***HC*** is a vector of household‐level characteristics including household size, whether the household has television, whether the household has flush toilet, whether the household has a refrigerator, whether the household has a washing machine, and whether the household has a car/motorcycle. *Urban*_*j*_ is a dummy indicating individual *j*'s residence in urban areas, *Division*_*j*_ is division fixed effect, and *ζ*_*j*_ is idiosyncratic error term. Errors are clustered at the division level. Regressions are separately estimated for all individuals, current tobacco users, and tobacco nonusers. The exposure analyses set the premise for DD analysis of household‐level tobacco consumption.

### 2.5 DD model

Finally, we estimate a DD model using HIES 2011–2012, 2013–2014, and 2015–2016 data. We include the 2012 data in DD analysis to control for pretreatment trends. Based on the results from exposure to antitobacco campaign analysis, we assume that the treatment coverage, that is, the reach of the progress in *W*_2_ measure of MPOWER in 2014, was greater for *higher than primary education* households. In this way, the *higher than primary education* group can be considered as the treatment group, and the *primary or no education* group can be considered as the control group in a DD specification as in Equation 3. Unlike the traditional DD setup, the control group is not fully untreated in this setting; rather, the treatment intensity (i.e., exposure to antitobacco information) for the control group is lower than that for the treatment group. Similar DD design was used by Duflo (2001) to estimate the returns of education by exploiting the regional differences in the number of schools (per 1,000 children) constructed during the school construction program in Indonesia.

(3) $$\text{Tobacc}o_{i}=\beta_{0}+\beta_{1}\mathrm{Edu}c_{i}+\sum_{t=2}^{3}\gamma_{t}\mathrm{Yea}r_{t,i}+\beta_{2}\mathrm{Edu}c_{i}*\mathrm{Pos}t_{i}+\beta_{3}\text{Urba}n_{i}+X_{i}\beta_{4}+\text{Divisio}n_{i}+\varepsilon_{i}\;$$

where *Tobacco*_*i*_ is the dummy indicating household's tobacco consumption. *Educ*_*i*_ is a dummy that takes the value 1 if the highest education level of household *i* is *higher than primary education*, and 0 if that is *primary or no education.* *Year*_*t*_ are dummies that take the value 1 if the year is *t* (2012, 2014, or 2016) and 0 otherwise, and *Post*_*i*_ is a dummy that takes the values 1 if the year is 2016. ***X*** is a vector of household‐level characteristics as in Equation 1. *Urban*_*i*_ indicates whether household *i* is located in urban areas, *Division*_*i*_ is division fixed effect, and *ε*_*i*_ is the idiosyncratic error term. Standard errors are clustered at the division level. Equation 1 is separately estimated for the full sample and urban and rural subsamples. The coefficient of interest in this specification is that of the interaction term of *Educ*_*i*_ and *Post*_*i*_, *β*_*2*_, which can be expressed as follows:

(4) $$\left[E\left(\text{Tobacc}o_{i}|\mathrm{Edu}c_{i}=1,\mathrm{Pos}t_{i}=1\right)-E\left(\text{Tobacc}o_{i}|\mathrm{Edu}c_{i}=1,\mathrm{Pos}t_{i}=0\right)\right]-\left[E\left(\text{Tobacc}o_{i}|\mathrm{Edu}c_{i}=0,\mathrm{Pos}t_{i}=1\right)-E\left(\text{Tobacc}o_{i}|\mathrm{Edu}c_{i}=0,\mathrm{Pos}t_{i}=0\right)\right]=\beta_{2}\;$$

The *higher than primary education* group is a selected group that may be different from the *primary or no education* group in observable and unobservable ways. For *β*_2_ to be causally interpretable, the identifying assumption is that the two groups would have parallel trends in tobacco‐use prevalence had there been no treatment. It requires the magnitude of the selection bias to be time invariant, that is, the reasons why the *higher than primary education* group notices more antitobacco information do not change over time. And time trends of tobacco‐use prevalence of the *primary or no education* and *higher than primary education* groups do not change in the absence of treatment; that is, the gap in tobacco‐use prevalence between the less educated and the more educated should be the same had they not have differential exposure to antitobacco information.

Under these assumptions, a negative and statistically significant estimate of *β*_*2*_ implies a greater decrease in the share of tobacco‐user households post 2014 for the *higher than primary education* household group. We visually check the parallel trend conditions by plotting the share of tobacco‐user households over time (2012, 2014, and 2016) for the two household groups of different educational attainment. We also run a model adding a squared term of the log of household income per capita and another model including income per capita quintile dummies instead of a continuous log of income per capita. Neither of these two versions changes the original estimate of *β*_*2*_.

We check the robustness of our DD estimates by conducting two falsification tests. First, we consider 2012 as the placebo treatment time and exclude the actual posttreatment (i.e., 2016) observations. We run the DD specification using data for prior periods, for which *Post*_*i*_ takes the value 1 if the year is 2014 and 0 if the year is 2012. Second, we consider a placebo control group, households with a maximum adult (15+ age) education being *secondary or less*, *but higher than primary education*, and a new treatment group *higher than secondary education.* The original control group of *primary or no education* is excluded. From the 2014 GATS, we verify that the difference in treatment exposure between these two groups is significantly smaller than that of the original treatment and control groups. Hence, we should not get any meaningful treatment effect, that is, the difference in tobacco‐use outcomes across these new groups. We run the DD specification using *high school or above* households as treatment and *secondary or less* households as control groups. Insignificant estimates of *β*_*2*_ (i.e., estimate of *β*_*2*_ is not statistically different from zero) of these two specifications (placebo treatment time and placebo control group) would validate our original DD estimates.

Because education and income are closely associated, we further explore the interaction between the two in assessing the prevalence gap across educational groups. First, to limit the variation in income, we created subsamples excluding or including certain household groups based on income per capita quintiles. We estimate the DD model for four subsamples: top four quintiles, bottom four quintiles, middle three quintiles, and top three quintiles to check whether our results are robust across these subgroups. Next, we allow income to vary within the educational groups and estimate a triple difference (DDD) model, where the third dimension is income (in continuous form). The treatment effect thus varies across income levels in the DDD specification. We also check the robustness of our results by further classifying the treatment group (*higher than primary education*) in two categories—*secondary or less* and *high school and above* and estimating the DD model with two treatment groups.

## 3 RESULTS

**FIGURE 2:**
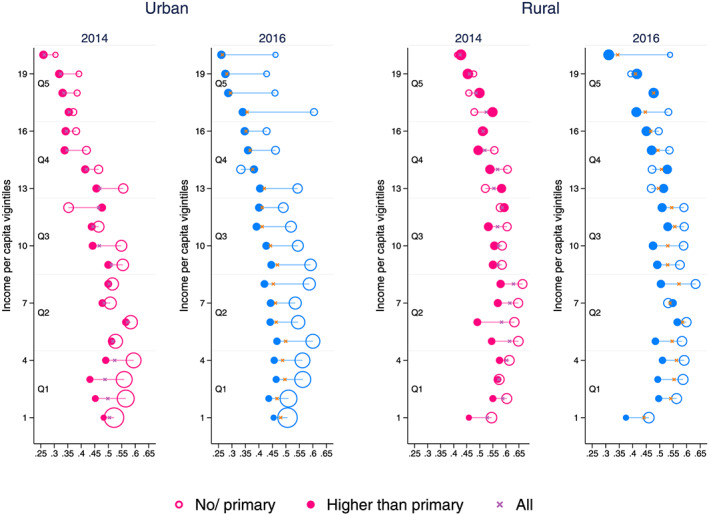
Tobacco use prevalence by income per capita (vigintiles) and difference in prevalence between the two educational groups. The horizontal axis shows the share of households consuming tobacco (%). The size of the hollow bubbles represents the share of “no/primary education” households in respective vigintiles. The size of solid bubbles represents the share of “higher than primary education” households in respective vigintiles [Colour figure can be viewed at wileyonlinelibrary.com]

From 2014 to 2016, the share of tobacco‐user households in Pakistan decreased from 51.5% to 45.5%. The decrease was similar (around 5 to 6 percentage points) across different income per capita quintiles. However, the decrease varies widely across households of different educational attainment. For the *primary or no education* households, tobacco‐use prevalence decreases by 4 percentage points (from 56% in 2014), whereas it decreases by 6.5 percentage points (from 48% in 2014) for the *higher than primary education* households. This difference between the two educational groups is evident across the household's economic status (income per capita) and urban and rural residence. Almost at every income level, in both urban and rural areas, the gap in tobacco‐use prevalence between the two educational groups widens in 2016 (Figure 2), suggesting a differential impact of progress made in tobacco control in 2014 across the educational groups.

Table 1 shows the same phenomena as in Figure 2 in a more rigorous way. It shows that compared to 2014, household‐level tobacco‐use prevalence decreased by 3.1 percentage points for all households in 2016 but the magnitude varied by households' level of educational attainment. After controlling for income and sociodemographic characteristics, household‐level tobacco‐use prevalence among the *higher than primary education* group decreased by 3.6 percentage points from 2014 to 2016, whereas the change for the *primary or no education* group was not significantly different from zero.

**TABLE 1:** Comparison of LPM results between two groups

	All	Primary or no education	Higher than primary education
Year dummies			
Year_2016_	−0.031^**^ (−0.060, −0.002)	−0.019 (−0.054, 0.016)	−0.036^**^ (−0.069, −0.003)
Log income per capita	−0.082^***^ (−0.098, −0.066)	0.009 (−0.011, 0.029)	−0.070^***^ (−0.086, −0.053)^†††^
Share of adult household members	0.001^***^ (0.001, 0.002)	0.002^***^ (0.001, 0.002)	0.002^***^ (0.001, 0.002)
Proportion of male among adults	0.003^***^ (0.003, 0.004)	0.003^***^ (0.003, 0.004)	0.003^***^ (0.002, 0.003)
Share of elderly 65+	0.000 (−0.000, 0.001)	0.001^**^ (0.000, 0.002)	0.000 (−0.001, 0.001)
Female‐headed household	−0.207^***^ (−0.242, −0.172)	−0.308^***^ (−0.347, −0.269)	−0.167^***^ (−0.209, −0.125)^†††^
Household size dummies			
3 to 5	0.044^***^ (0.026, 0.061)	0.106^***^ (0.077, 0.136)	0.067^***^ (0.044, 0.090)
6 to 9	0.093^***^ (0.069, 0.116)	0.178^***^ (0.145, 0.211)	0.131^***^ (0.102, 0.161)
10 and more	0.179^***^ (0.145, 0.212)	0.246^***^ (0.202, 0.289)	0.244^***^ (0.206, 0.282)
Urban	−0.090^***^ (−0.119, −0.061)	−0.043^***^ (−0.071, −0.016)	−0.083^***^ (−0.115, −0.051)^†††^
Constant	0.893^***^ (0.786, 0.999)	0.191^**^ (0.033, 0.350)	0.736^***^ (0.617, 0.854)^†††^
Division fixed effect	Yes	Yes	Yes
Observations	42,091	14,201	27,890
*R* squared	0.135	0.176	0.118

*Note*: 95% confidence intervals in parentheses. In addition, we denote statistically significantly different effect for *primary or no education* relative to *higher than primary education* for the year dummy, log of per capita income, share of adult household members, proportion adult male, share of elderly, female‐headed household, and urban by dagger symbols.

Abbreviation: LPM, linear probability model.

^***^ *p* < 0.01.

^**^ *p* < 0.05.

^*^ *p* < 0.1.

^†††^ At 1% level.

^††^ At 5% level.

^†^ At 10% level using a Chow test.

Table 2 shows that there were significant differences in exposure to antitobacco campaigns between individuals with *higher than primary education* and *primary or no education.* On average, individuals with higher than primary education were, respectively, 14.4 percentage points and 6.4 percentage points more likely to notice anticigarette and antismokeless tobacco information in 2014. For both current tobacco‐user and nonuser categories, *higher than primary education* group was more likely to notice antitobacco information. Individuals with *higher than primary education* were also more likely to notice health warnings on tobacco product packages. Hence, the reach of the national antitobacco campaign was greater for the *higher than primary education* group. In other words, the treatment coverage was greater for the *higher than primary education* group than the *primary or no education* group.

**TABLE 2:** Adjusted differences in antitobacco campaign exposure between individuals with higher than primary and primary or no education

	All	Current tobacco user	Tobacco nonuser
Noticed anticigarette information			
Anywhere	0.144^***^ (0.112, 0.175)	0.126^***^ (0.069, 0.183)	0.145^***^ (0.113, 0.178)
Radio/TV	0.114^***^ (0.088, 0.140)	0.109^***^ (0.048, 0.170)	0.114^***^ (0.088, 0.141)
Billboards	0.053^***^ (0.033, 0.073)	0.03 (−0.011, 0.070)	0.057^***^ (0.035, 0.078)
Noticed antismokeless tobacco information			
Anywhere	0.064^***^ (0.032, 0.095)	0.100^*^ (−0.020, 0.221)	0.060^***^ (0.029, 0.091)
Radio/TV	0.057^***^ (0.004, 0.030)	−0.003 (−0.001, 0.072)	0.060^***^ (0.001, 0.031)
Noticed health warning on			
Cigarette packages	0.071^***^ (0.037, 0.104)	0.041 (−0.032, 0.114)	0.084^***^ (0.048, 0.119)
Smokeless tobacco products	0.013^**^ (0.000, 0.025)	0.085^**^ (0.018, 0.153)	0.010 (−0.005, 0.025)

*Note*: 95% confidence intervals in parentheses. Adjusted differences account for gender, age, marital status, occupation, household size, whether the household has television, whether the household has flush toilet, whether the household has refrigerator, whether the household has washing machine, whether the household has car/motorcycle, urban/rural residence, and administrative division fixed effects. Standard errors for adjusted differences are clustered at the division level. For anticigarette information and health warning on cigarette packages, tobacco user refers to daily and occasional (less than daily) smokers, and tobacco nonuser refers to former and never smokers. For antismokeless tobacco information and health warning on smokeless tobacco products, tobacco user refers to daily and occasional users of smokeless tobacco, and tobacco nonuser refers to former and never smokeless tobacco‐users.

^***^ *p* < 0.01.

^**^ *p* < 0.05.

^*^ *p* < 0.1.

**FIGURE 3:**
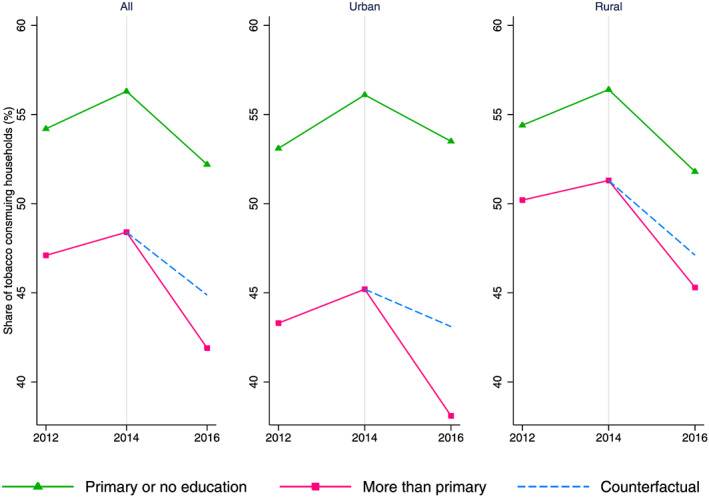
Trends in share of tobacco‐consuming households by education level [Colour figure can be viewed at wileyonlinelibrary.com]

Figure 3 illustrates the required parallel trend condition for the difference in differences analysis. The counterfactual line in the figure is drawn assuming the gap between *primary or no education* and *higher than primary education* in 2014 would prevail in 2016, in the absence of MPOWER interventions. It shows that during the pretreatment period (i.e., 2012 to 2014), the trends in the share of tobacco‐consuming households were fairly parallel across the *primary or no education* and *higher than primary education* groups. This gives credence to the validity of our identification strategy.

Table 3 shows the main results of our analysis. The three columns are three different regressions for all households (full sample) and urban and rural subsamples. The results show that subject to the sociodemographic controls, greater exposure to antitobacco measures led to a 3.6 percentage point decrease in the share of tobacco‐consuming households for the *higher than primary education* group compared to the *primary or no education* group. The effect size was very similar for the urban subsample, while not statistically significant for the rural subsample.

**TABLE 3:** Difference‐in‐differences regression results

	All	Urban	Rural
Education*Post	−0.036^**^ (−0.065, −0.008)	−0.038^**^ (−0.075, −0.001)	−0.017 (−0.055, 0.021)
Education	−0.103^***^ (−0.122, −0.083)	−0.111^***^ (−0.136, −0.086)	−0.109^***^ (−0.131, −0.088)
Year Dummies			
Year_2014_	0.024^**^ (0.001, 0.048)	0.021 (−0.005, 0.048)	0.031^*^ (−0.001, 0.064)
Year_2016_	0.019 (−0.015, 0.053)	0.024 (−0.023, 0.070)	0.005 (−0.036, 0.046)
Log income per capita	−0.050^***^ (−0.066, −0.034)	−0.070^***^ (−0.084, −0.057)	−0.017 (−0.037, 0.004)
Share of adults	0.002^***^ (0.001, 0.002)	0.001^***^ (0.001, 0.002)	0.002^***^ (0.002, 0.002)
Proportion of male among adults	0.003^***^ (0.003, 0.004)	0.003^***^ (0.003, 0.003)	0.003^***^ (0.003, 0.004)
Share of elderly 65+	0.000 (−0.000, 0.001)	−0.000 (−0.001, 0.000)	0.001^**^ (0.000, 0.001)
Female‐headed household	−0.222^***^ (−0.255, −0.190)	−0.154^***^ (−0.181, −0.128)	−0.292^***^ (−0.316, −0.268)
Household size dummies			
3 to 5	0.074^***^ (0.055, 0.092)	0.050^***^ (0.018, 0.082)	0.097^***^ (0.069, 0.126)
6 to 9	0.139^***^ (0.111, 0.167)	0.099^***^ (0.062, 0.136)	0.181^***^ (0.145, 0.218)
10 and more	0.234^***^ (0.201, 0.268)	0.205^***^ (0.157, 0.253)	0.265^***^ (0.219, 0.311)
Urban	−0.075^***^ (−0.103, −0.048)	‐	‐
Constant	0.627^***^ (0.517, 0.737)	0.802^***^ (0.691, 0.912)	0.337^***^ (0.201, 0.472)
Division fixed effect	Yes	Yes	Yes
Observations	57,875	29,073	28,802
*R* squared	0.135	0.121	0.144

*Note*: 95% confidence intervals in parentheses. *Year*_2012_ is the reference group for year dummies. Household size of 2 or less is the reference group for household size dummies.

^***^ *p* < 0.01.

^**^ *p* < 0.05.

^*^ *p* < 0.1.

Two different falsification tests are presented to check the validity of our method. The top panel in Table 4 shows the results for a placebo treatment time. Confining the sample period to 2012 and 2014 and assigning 2012 as the year of treatment, we find no effect on household's probability of tobacco use (i.e., the share of households consuming tobacco). This holds true for the full sample as well as for the urban and rural subsamples. The bottom panel of Table 4 shows the results of the placebo control group. Here the sample is confined to *higher than primary education* households. Among these households, *secondary or less education* is considered as the control group, and *high school and above* is considered as the treatment group. The adjusted difference in antitobacco campaign exposure between individuals with *high school and above education* and *secondary or less but higher than primary education* is statistically insignificant and much lower (4.1 percentage points for anticigarette and 1.5 percentage points for antismokeless tobacco) than that (14.4 percentage points for anticigarette and 6.4 percentage points for antismokeless tobacco) between the original treatment (higher than primary) and control (primary or no education) groups. This exercise also generates no significant treatment effects. The DD identification strategy thus passes both falsification tests.

**TABLE 4:** Falsification test results

	All	Urban	Rural
Placebo treatment time			
Education*Post	0.002 (−0.025, 0.029)	−0.009 (−0.054, 0.035)	0.005 (−0.030, 0.040)
Education	−0.106^***^ (−0.134, −0.079)	−0.109^***^ (−0.140, −0.079)	−0.112^***^ (−0.145, −0.078)
Year dummies			
Year_2014_	0.022 (−0.014, 0.057)	0.028 (−0.026, 0.082)	0.028 (−0.012, 0.067)
Constant	0.574^***^ (0.444, 0.705)	0.848^***^ (0.699, 0.997)	0.301^***^ (0.164, 0.437)
Division fixed effect	Yes	Yes	Yes
Observations	33,645	12,924	20,721
*R* squared	0.137	0.127	0.141
Placebo control group			
Education*Post	−0.014 (−0.034, 0.005)	−0.005 (−0.026, 0.017)	−0.019 (−0.058, 0.021)
Education	−0.128^***^ (−0.141, −0.116)	−0.141^***^ (−0.156, −0.125)	−0.111^***^ (−0.131, −0.090)
Year dummies			
Year_2014_	0.023^**^ (0.004, 0.043)	0.017 (−0.005, 0.039)	0.035^**^ (0.001, 0.068)
Year_2016_	−0.007 (−0.046, 0.031)	−0.014 (−0.060, 0.033)	−0.004 (−0.050, 0.041)
Constant	0.348^***^ (0.242, 0.454)	0.416^***^ (0.284, 0.549)	0.153^**^ (0.007, 0.299)
Division fixed effect	Yes	Yes	Yes
Observations	37,759	23,392	14,367
*R* squared	0.127	0.127	0.118

*Note*: Placebo treatment time is 2012, which affects household tobacco consumption outcome of 2014. Placebo control group consists of households for which the highest level of education is secondary or less but higher than primary education, and the treatment group consists of households for which the highest level of education is high school and above; 95% confidence intervals in parentheses. Year_2012_ is the reference group for year dummies. Control variables not reported here include log income per capita, share of adults, proportion of male among adults, share of elderly 65+, sex of household head, household size, and household's location (urban/rural).

^***^ *p* < 0.01.

^**^ *p* < 0.05.

^*^ *p* < 0.1.

**FIGURE 4:**
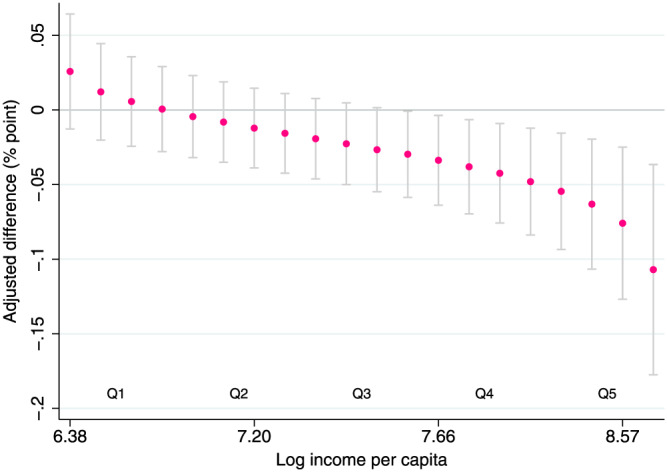
Adjusted differences in change in tobacco‐use prevalence between the two educational groups at different income levels. We calculate the average log income per capita at each vigintile and estimate the adjusted difference at that income level. The vertical lines represent 95% confidence intervals. Confidence intervals are estimated using the Delta method [Colour figure can be viewed at wileyonlinelibrary.com]

The results of the income subgroup analysis are presented in Table 5. Though the differences between the two educational groups were slightly higher for the top three quintiles and marginally lower for the bottom four quintiles, the main results remained very similar to our original estimate. The treatment effect for the middle three quintiles was almost the same as the original estimate. The triple difference estimate further suggests that the effect size was larger for households with higher per capita income and the effect was not statistically significant for the poorest households (Figure 4).

**TABLE 5:** Difference‐in‐differences estimates by income subgroups

	Top four	Bottom four	Middle three	Top three
Education*Post	−0.042^***^ (−0.072, −0.012)	−0.030^**^ (−0.056, −0.004)	−0.036^***^ (−0.062, −0.010)	−0.052^***^ (−0.086, −0.018)
Education	−0.103^***^ (−0.125, −0.082)	−0.111^***^ (−0.131, −0.091)	−0.107^***^ (−0.130, −0.085)	−0.101^***^ (−0.124, −0.077)
Year Dummies				
Year_2014_	0.022^*^ (−0.001, 0.045)	0.029^**^ (0.001, 0.056)	0.028^**^ (0.002, 0.055)	0.018 (−0.004, 0.040)
Year_2016_	0.023 (−0.013, 0.059)	0.012 (−0.024, 0.048)	0.021 (−0.017, 0.058)	0.030^*^ (−0.006, 0.065)
Division fixed effect	Yes	Yes	Yes	Yes
Observations	47,364	45,432	34,921	35,942
*R* squared	0.140	0.126	0.129	0.140

*Note*: 95% confidence intervals in parentheses. “Top 4” includes all households but those in the first quintile. “Bottom 4” includes all households but those in the fifth quintile. “Middle 3” includes households of the second, third, and fourth quintiles. “Top 3” includes households of the third, fourth, and fifth quintiles. Year_2012_ is the reference group for year dummies. Control variables not reported here include log income per capita, share of adults, proportion of male among adults, share of elderly 65+, sex of household head, household size, and household's location (urban/rural).

^***^ *p* < 0.01.

^**^ *p* < 0.05.

^*^ *p* < 0.1.

Finally, Table 6 shows the DD results with two treatment groups. Tobacco‐use prevalence of the *high school or above* group decreased by 3.7 percentage points more compared to the *primary or no education* group in the full sample, whereas the 2.2 percentage point difference between the *secondary or less* and *primary or no education* group was not statistically significant. For the urban subsample, the differences were significant for both *secondary or less* and *high school or above* groups, though the size was marginally greater for the latter. Like our original estimates, none of the differences were statistically significant for the rural subsample.

**TABLE 6:** Difference‐in‐differences results with two treatment groups

	All	Urban	Rural
Education1*Post	−0.022 (−0.051, 0.007)	−0.032^*^ (−0.068, 0.004)	−0.009 (−0.052, 0.034)
Education2* Post	−0.037^**^ (−0.067, −0.007)	−0.037^*^ (−0.077, 0.003)	−0.029 (−0.071, 0.013)
Education1	−0.067^***^ (−0.090, −0.044)	−0.062^***^ (−0.090, −0.033)	−0.080^***^ (−0.105, −0.055)
Education2	−0.196^***^ (−0.218, −0.173)	−0.201^***^ (−0.229, −0.173)	−0.189^***^ (−0.215, −0.163)
Year Dummies			
Year_2014_	0.023^*^ (−0.001, 0.047)	0.018 (−0.008, 0.044)	0.031^*^ (−0.001, 0.064)
Year_2016_	0.013 (−0.022, 0.048)	0.019 (−0.027, 0.066)	0.004 (−0.038, 0.045)
Division Fixed Effect	Yes	Yes	Yes
Observations	57,875	29,073	28,802
*R* squared	0.146	0.134	0.149

*Note*: 95% confidence intervals in parentheses. Education1 refers to *secondary or less*, and Education2 refers to *high school or above.* Year_2012_ is the reference group for year dummies. Control variables not reported here include log income per capita, share of adults, proportion of male among adults, share of elderly 65+, sex of household head, household size, and household's location (urban/rural).

^***^ *p* < 0.01.

^**^ *p* < 0.05.

^*^ *p* < 0.1.

## 4 DISCUSSION

### 4.1 Main findings

We find that progress in MPOWER implementation in 2014 in Pakistan was associated with 3.1 percentage point reduction in household‐level tobacco‐use prevalence from 2014 to 2016. However, the reduction in tobacco use varied widely across households of different educational attainment. Higher than primary education households experienced 3.6 percentage point reduction in tobacco‐use prevalence, whereas primary or no education households did not experience any statistically significant change in tobacco‐use prevalence. From the 2014 GATS, we see that exposure to antitobacco information varied widely across educational groups. We find that individuals who attain higher than primary education were 14.4 percentage points more likely to notice anticigarette information than the individuals with primary or no education. We exploit this variation in treatment exposure to estimate the potential causal impact of progress in MPOWER implementation on household tobacco use in Pakistan. We find that because of differential coverage in MPOWER implementation, household‐level tobacco‐use prevalence decreased by 3.6 percentage points for the *higher than primary education* group compared to that of the *primary or no education* group.

### 4.2 Consistency with previous literature

There is not much evidence on the differential impacts of mass media campaigns on tobacco consumption by different SES in the developing countries. A systematic meta‐analysis of the effectiveness of media campaigns in promoting smoking cessation in low SES populations in the United States, Canada, Australia, and Western European nations finds that such campaigns are generally less, sometimes equally, and rarely more effective on low SES populations compared to the more advantaged groups (Niederdeppe et al., 2008). A more recent meta‐analysis finds that population‐based media campaigns, in general, have a lower impact on low SES populations (Hill, Amos, Clifford, & Platt, 2014). Our paper extends this literature in a developing country setting and finds a greater decrease in household‐level tobacco‐use prevalence for the higher educated group. The results are attributable to differential exposure of the higher educated group to health warnings and mass media antitobacco campaigns. These results are largely in line with the existing evidence on the differential effectiveness of mass media campaigns and health warnings across different SES groups in the developed countries.

### 4.3 Cautionary remarks on empirical analysis

We estimate the average treatment effect on the treated (ATT), which is the mean difference in outcome (tobacco‐use prevalence) between the treated and the control groups. We define treatment as greater exposure to MPOWER interventions. During the treatment period (2012 to 2014), *M*, *O*, *E*, and *W*_2_ measures of the MPOWER package show progress in implementation. We argue that the coverage of *O* (cessation) is very small in the Pakistan context and the progress in *M* (monitoring) and *E* (advertisement ban) does not have differential exposure across educational groups. We get evidence from the 2014 GATS that the reach of antitobacco information, that is, the coverage of *W*_2_ measure, varied widely across educational groups. Hence, differences in coverage of MPOWER implementation are primarily attributable to the differential reach of antitobacco information across educational groups. Though the MPOWER package is implemented at the national level and thus does not allow for regional variations in treatment, the difference in program coverage across socioeconomic (e.g., educational attainment) groups could be exploited to estimate ATT. In this way, our DD analysis is slightly different from the traditional DD approach and should be interpreted with some caution.

First, in a traditional DD setting, the treatment group is exposed to certain interventions, and the control group does not. In our case, both *primary or no education* and *higher than primary education* households are supposed to get exposed to national‐level MPOWER implementation. However, we observe that coverage of MPOWER differs significantly across the two groups. Hence, our estimates are derived from MPOWER coverage differential, and not MPOWER implementation in general. Second, our estimation approach relies heavily on the observed differences in noticing antitobacco information across educational groups, and not on how individuals of different education level may process antitobacco information to decide on quitting tobacco consumption. It is beyond the scope of this paper to analyze how behavioral differences across educational groups could impact tobacco use. Such analysis would at the least require similar treatment coverage across educational groups and then analyzing the difference in outcome between the groups, which is a very different question than that we ask in this paper.

Third, the coverage differential, attributable to educational attainment, may emanate from various sources. Higher educated individuals may have a latent ability to notice more antitobacco information when available. The content of the antitobacco campaign products (e.g., advertisements and flyers) may be difficult to understand for individuals of lower educational attainment. But without MPOWER interventions, these differences across educational groups do not realize; that is, the decision to continue or quit tobacco‐use does not change. The inherent differences in tobacco‐use decisions across *higher than primary education* and *primary or no education* groups are evident in the pretreatment period gap in tobacco‐use prevalence between the two groups. We do not analyze what explains the gap between the two education groups. Rather, we analyze how the gap changes in the posttreatment period because of the treatment coverage differential. We assume that the behavioral differences across educational groups and other attributes (e.g., price and income elasticities) related to tobacco‐use decision do not change for respective groups over time (during the study period) and thus canceled out by the within‐group across time difference. It also implies that given the behavioral differences (if any) across the groups, the gap in tobacco‐use prevalence between the two groups would prevail without any differential coverage of MPOWER intervention.

### 4.4 Other limitations

A limitation of our analysis is that we could not determine whether the content of the antitobacco messaging, or the campaign coverage strategy, or both are accountable for differential exposure to antitobacco information across different educational groups. We only observe the exposure differences in the data and could not infer the qualitative aspects of campaign effectiveness. Therefore, we cannot deduce whether less exposure or less effectiveness of antitobacco messaging contributes more in widening the gap in tobacco‐use prevalence across households of different SES. Mixed methods research, entailing both quantitative and qualitative aspects of tobacco control measures, may enrich our understanding in this regard. Another limitation is that we assume no exposure difference across educational groups in relation to advertising bans. However, advertising bans, in general, have a negative association with tobacco consumption in developing countries (Blecher, 2008). It may differentially affect different education groups. Hence, any differential impact emanated from behavioral differences across educational groups is also contained in our treatment effect estimates.

### 4.5 Concluding remarks

Despite the limitations, our analyses provide evidence that progress in tobacco control through MPOWER package implementation in a developing country can reduce tobacco‐use prevalence (at the household level). The basis of this conclusion is the differential coverage of MPOWER implementation across educational groups. Our findings further suggest that tobacco control measures may not deliver consistent outcomes across households with different levels of educational attainment. The reach of national‐level antitobacco campaigns can vary widely across socioeconomic groups, resulting in different outcomes (i.e., tobacco‐use prevalence) in the postintervention period. Policymakers may consider this issue while developing population‐level policies of inclusive and effective tobacco control measures.

## FUNDING INFORMATION

No funding was received for this research.

## CONFLICT OF INTEREST

None.

## DISCLAIMER

The findings and conclusions in this report are those of the authors and do not necessarily represent the official position of the Centers for Disease Control and Prevention.
